# Survival and neurologic outcome after traumatic out-of-hospital cardiopulmonary arrest in a pediatric and adult population: a systematic review

**DOI:** 10.1186/cc11410

**Published:** 2012-07-06

**Authors:** Jörn Zwingmann, Alexander T Mehlhorn, Thorsten Hammer, Jörg Bayer, Norbert P Südkamp, Peter C Strohm

**Affiliations:** 1 Department of Orthopedic Surgery and Traumatology, Freiburg University Hospital, IMS Robert-Koch-Str. 1, Freiburg, D-79106, Germany

## Abstract

**Introduction:**

This systematic review is focused on the in-hospital mortality and neurological outcome of survivors after prehospital resuscitation following trauma. Data were analyzed for adults/pediatric patients and for blunt/penetrating trauma.

**Methods:**

A systematic review was performed using the data available in Ovid Medline. 476 articles from 1/1964 - 5/2011 were identified by two independent investigators and 47 studies fulfilled the requirements (admission to hospital after prehospital resuscitation following trauma). Neurological outcome was evaluated using the Glasgow outcome scale.

**Results:**

34 studies/5391 patients with a potentially mixed population (no information was found in most studies if and how many children were included) and 13 paediatric studies/1243 children (age ≤ 18 years) were investigated. The overall mortality was 92.8% (mixed population: 238 survivors, lethality 96.7%; paediatric group: 237 survivors, lethality 86.4% = p < 0.001).

Penetrating trauma was found in 19 studies/1891 patients in the mixed population (69 survivors, lethality: 96.4%) and in 3 pediatric studies/91 children (2 survivors lethality 97.8%).

44.3% of the survivors in the mixed population and 38.3% in the group of children had a good neurological recovery. A moderate disability could be evaluated in 13.1% in the mixed population and in 12.8% in children. A severe disability was found in 29.5% of the survivors in the mixed patients and in 38.3% in the group of children. A persistent vegetative state was the neurological status in 9.8% in the mixed population and in 10.6% in children.

For each year prior to 2010, the estimated log-odds for survival decreased by 0.022 (95%-CI: [0.038;0.006]). When jointly analyzing the studies on adults and children, the proportion of survivors for children is estimated to be 17.8% (95%-CI: [15.1%;20.8%]). The difference of the paediatric compared to the adult proportion is significant (p < 0.001).

**Conclusions:**

Children have a higher chance of survival after resuscitation of an out-of-hospital traumatic cardiac arrest compared to adults but tend to have a poorer neurological outcome at discharge.

## Introduction

Modern emergency medical services (EMS) systems began in the 1960s to provide emergency medical care in the community in industrialized countries. Historically, the aim of EMS was responding to any life-threatening emergency. Later on some of the goals were also providing rapid medical response and treatment to patients suffering out-of-hospital cardiopulmonary arrest (OHCA) and to victims of vehicular trauma.

A large group of patients do not survive traumatic OHCA despite appropriate, immediate, and extensive efforts by bystanders and EMS personnel. These patients present challenges to the system, because prehospital resuscitative efforts are labor- and resource-intensive, as are these efforts when continued in the emergency department (ED). It is usually assumed that trauma victims with cardiac arrest in the prehospital setting have a dismal prognosis, leading physicians to consider cardiopulmonary resuscitation in traumatic cardiac arrest as futile [1]. The epidemiology of mortality following injury suggests that 34% of traumatic deaths occur before hospital arrival [2]. Despite advances in medical care, survival rates of 0% to 2% have been reported for patients with blunt trauma who arrive at a trauma center with no signs of life [3-7]. Unfortunately, many of these survivors suffer from severe permanent neurological disability [6,8].

The National Association of EMS Physicans/American College of Surgery Committees on Trauma produced guidelines in 2003 about withholding or termination of resuscitation efforts in OHCA [7]. According to these guidelines, it is suggested not to resuscitate patients with blunt trauma and OHCA or patients with penetrating trauma who have no detectable pulse, are apneic and without signs of life, and accordingly to pronounce death at the scene of injury. On the other hand it has been proposed in some studies that some traumatic cardiac deaths following motor-vehicle collisions occur because of airway obstruction of the comatose patients [9,10]. Simple airway maneuvers and intubations could possibly prevent hypoxic cardiac arrest. Other early deaths following trauma might be due to exsanguination and might be prevented in some cases by external pressure on areas with visible, profuse blood loss. Since the publication of these guidelines, at least two articles have described improved survival rates [11,12] and one has described possible deviation of the guidelines [11]. On the other hand one recent article supported the current guidelines regarding the withholding or termination of resuscitation of patients with prehospital traumatic cardiopulmonary arrest (TCPA) [13].

However, despite the poor survival of pediatric trauma patients receiving cardiopulmonary resuscitation (CPR) (1.5% to 25%), cessation or withholding of CPR after traumatic injury in a child is a difficult and emotionally charged issue [14-20]. Clear-cut resuscitation guidelines for children requiring CPR after traumatic injury do not exist to our knowledge. A review suggested that almost 22% of cardiac arrests in children are trauma associated, rendering this one of the most common causes of pre-hospital cardiac arrest and death between the ages of 1 to 16 years ([5,21,22].

However, no strong evidence is available for the mortality in OHCA. Hence, the aim of this study was to summarize and analyze the best available data on mortality and survivor outcome after traumatic OHCA by performing a systematic literature review.

## Materials and methods

**Figure 1:**
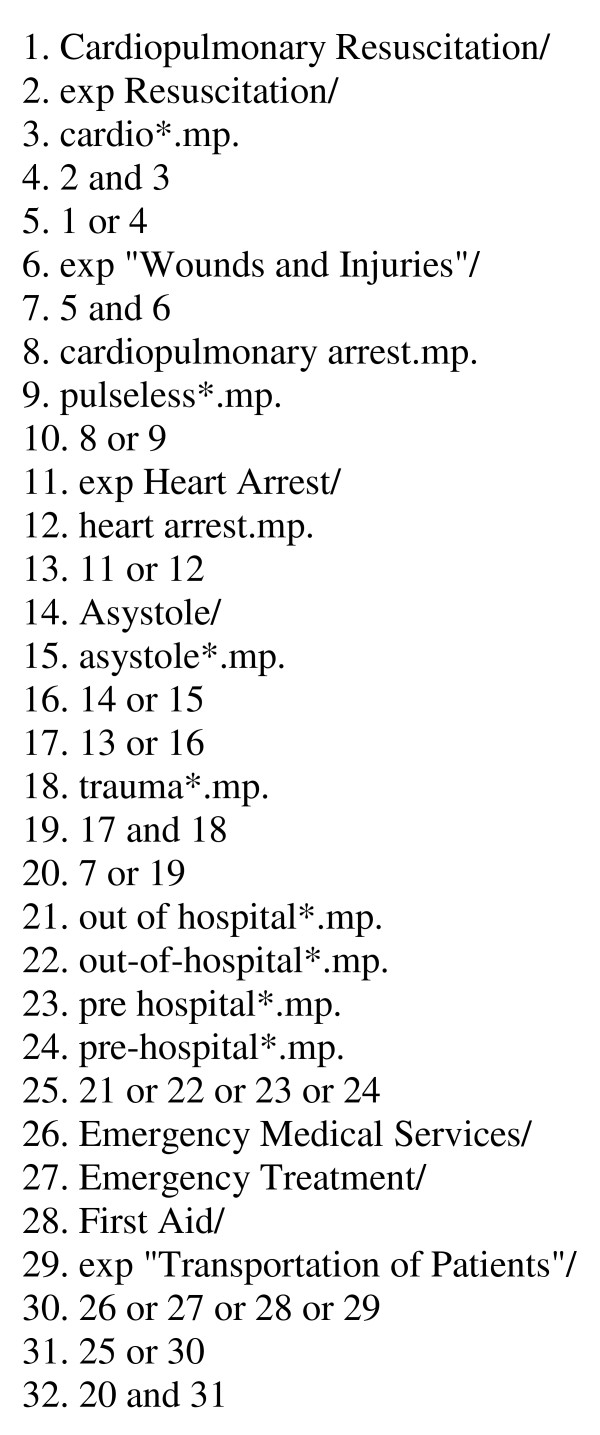
**Search terms and the strategy used to search the medical databases**.

For this systematic review, an Ovid Medline-based literature search was performed to identify any published clinical studies on mortality after out-of-hospital resuscitation of patients suffering a trauma, including the following databases: MEDLINE, MEDLINE preprints, EMBASE, CINAHL, Life Science Citations, British National Library of Health, Cochrane Central Register of Controlled Trials (CENTRAL). The literature search period was from January 1964 to May 2011. The search was performed on 15 May 2011 using the strategy given in Figure 1. Specific attention was placed on identifying studies that describe the neurological outcome of survivors.

**Figure 2:**
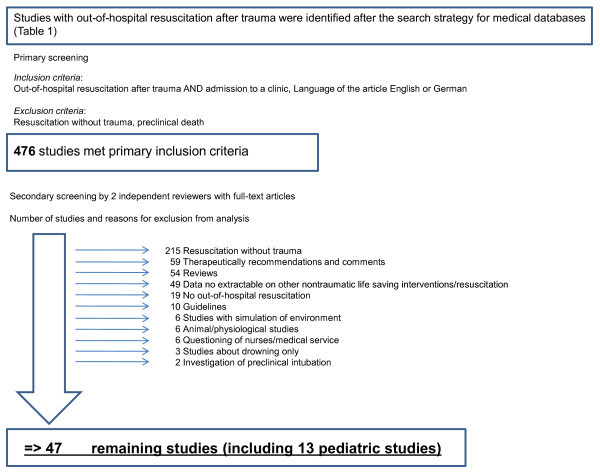
**Search strategy and criteria used for exclusion of studies**. Of 476 studies found, 429 had to be excluded from analysis for the reasons demonstrated, leaving 47 remaining studies (including 13 pediatric studies) for the final analysis.

A total of 476 clinical studies were identified and all abstracts were evaluated on screening by two independent reviewers. The only studies included were those reporting patients who received preclinical resuscitation after sustaining trauma, and who were admitted to hospital. The level of evidence was categorized according to the definition given by the Oxford Centre for Evidence-based Medicine published by Hanzlik [23]: 476 studies met these inclusion criteria and full-text articles of these studies were obtained for review. Of these, 429 studies had to be excluded from analysis for reasons demonstrated in Figure 2. The remaining 47 studies were included in the present analysis. Two independent reviewers systemically extracted data that included study characteristics and design, level of evidence, demographic and epidemiological parameters, number of included patients, mechanism of injury, neurological and outcome.

These studies were subdivided into those that were exclusively pediatric studies, and those in a general population in which children were not always explicitly excluded and therefore called a potentially mixed population. The average age in these groups was between 29 and 65 years. Some of the identified studies included children, but the vast majority were based on adults. Specific focus was placed on extracting data describing the trauma mechanism and analyzing the neurological outcome of survivors. To assess for the methodological quality of the neurological outcome the patients were divided into four groups using the Glasgow outcome scale with the following description [24]: I) persistent vegetative state: the patient exhibits no obvious cortical function; II) severe disability: the patient is conscious but disabled and depends upon others for daily support due to mental or physical disability or both; III) moderate disability: the patient is disabled but independent as far as daily life is concerned. The disabilities found include varying degrees of dysphasia, hemiparesis, or ataxia, as well as intellectual and memory deficits and personality changes, and IV) good recovery: the patient has resumed normal activities even though there may be minor neurological or psychological deficits.

The outcome parameters have been used in the studies included in this systematic review. The data were independently cross-checked with the original papers using a standard quality control procedure. Any differences of opinion between the original reviewer and quality control reviewer were resolved by discussion and referenced to the study paper. The data were analyzed using established statistical software.

To analyze the proportion of survivors from different studies, we used binomial response generalized linear models. This allowed analysis of the effect of factors that might influence the proportion. Specifically, we considered the year of publication, where 2010 was taken as a reference, the dimension of the study, and gender proportion. As the studies on children might differ from the studies on adults concerning these factors, we used the adult data to check for their influence, before incorporating factors into joint analysis of child and adult data. For statistical analyses we used statistical environment R (2.13.0) software (R foundation for Statistical Computing, Vienna, Austria).

## Results

### Characteristics of studies and patients included in the analysis

Forty-seven studies were included, describing the mortality of 6,634 patients admitted to hospital after resuscitation of TCPA. These articles were subdivided into 34 studies with a total of 5,391 patients comprising a potentially mixed population (most studies provide no information about how many children were included), and 13 studies concerning only pediatric populations, totaling 1,243 patients aged ≤ 8 years. The overall mortality was 92.8%. The mixed population of 5,391 patients had 238 survivors and therefore a mortality of 96.7%. In the solely pediatric group 237 survivors out of 1,243 patients were analyzed. This finding showed a significantly decreased mortality of 86.4%. In 15 mixed studies, information about the gender distribution was found for 1,832 male (77.2%) and 525 female patients. The gender distribution was described in five pediatric studies and comprised 166 boys (52.2%) and 150 girls (Tables 1 and 2).

**Table 1:** Summary of the mixed population studies included in the data analysis

First author	Year	Journal	N	Male/Female	Mean age	Survivors	Mortality %	BT	Survivors BT	Mortal BT %	PT	Survivors PT	Mort PT %	Outcome total	Good	Mod	Poor	Veget
Harner T	1981	The American Journal of Surgery	85			13	78											
Kenneth L	1982	J. Trauma	100			0	100	37	0	100	63	0	100					
Aprahamian C	1985	Annals of Emerg. Medi	95	75/20	29	3	96.8											
Wright S	1989	Annals of Emerg. Medi	47			0	100											
Hoyt D	1989	Arch Surg	48			4	83.3	25	0	100	23	4	82.6					
Lucian A	1992	J. Trauma	207			18	91	27	0	100	180	18	90					
Durham L	1992	J. Trauma	207			18	91.3	27	0	100	180	18	90					
Heinrich H	1992	Z. gesamte Inn. Med.	8			0	100											
Rosemurgy AS	1993	J. Trauma	138	90/48	36	0	100	96	0	100	42	0	100					
Fulton R	1995	Journal American College of Surg.	173			3	98.3											
Quintans-Rodriquez A	1995	Europ J of Emerg. Med	11			2	82											
Falcone R	1995	Air Med J	320	232/88	32.4	6	98.1	284	5	98.2	36	1	97.2	4		2		
Lawhon J	1995	The Journal of Tennessee Med. Assoc.	47	34/13		2	95.7	47	2	95.7				2			1	1
Pasquale M	1996	J. Trauma	106	76/30	41	3	97.2	85	2	97.7	21	1	95.3					
Margolin D	1996	J. Trauma	67	50/17	34	13	81	53	12	77.4	14	1	92.9	13	3	3	5	2
Cocanour S	1997	J. Trauma	11			0	100				11	0	100					
Kuisma M	1997	European Hear Journal	15			0	100											
Stratton S	1998	J. Trauma	879			9	99	382	5	98.7	497	4	99.2	9	3	1	2	3
Battistella F	1999	Arch Surg	604	471/133	36	16	97.4	304	4	98.7	300	2	96.0	16	9		7	
Coats TJ	2001	J. Trauma	39	35/4		4	89.7				39	4	89.7	4	3	1		
MacMillan D	2003	Prehospital Emergency Care	1	1/0		1	100				1	0	0	1		1		
Stockinger Z	2004	J. Am Coll Curg	588	492/96	34	22	96.3	194	12	93.8	341	3	99.1					
Fialka C	2004	J. Trauma	38	28/10	28	4	89.5							4	4			
Pickens J	2005	J. Trauma	184			14	92.4											
Bartolomeo S	2005	Prehosp Emerg Care	29			2	93.2	29	2	93.2				2			2	
Willis CD	2006	Injury	89			4	95	71	3	97	18	2	89	4	4			
Lockey D	2006	Annals of Emerg. Medi	909			68	92.5	542	27	95	114	9	96.1					
O'Brien E	2008	Prehosp Emerg Care	43	36/7	38	0	100	34	0	100	9	0	100					
David, J-S	2007	Crit Care Med	268	210/58	42	6	97.8											
Ashour A	2007	Emerg. Med. Austral.	11			0	100											
Kurimoto Y	2007	Surg. Today	1	1/0	57	1	0				1	1	0	1			1	
Corral E	2007	Resucitation	1	1/0		1	0				1	1	0	1	1			
Whalen W	2008	JEMS	1	0/1	65	0	100	1	0	100								
Franek O	2010	Resucitation	21			1	95.2											

Total			5,391	1,832/525	39.4	238	89.1	2,238	74	96.7	1,891	69	96.4	61	27	8	18	6

BT: blunt trauma; PT; penetrating trauma; Mort: mortality. Good, Mod, Poor and Veget indicate good, moderate, poor and vegetative outcomes.

**Table 2:** Summary of the pediatric studies included in the data analysis

Author	Year	Journal	N	Boys/Girls	Mean age	Survivors	Mort %	BT	Survivors BT	Mort BT%	PT	Survivors PT	Mort PT%	Outcome total	Good	Mod	Poor	Veget
Hazinski	1994	Annals of Emerg Med	38		5	1	97.4	38	1	97.4							1	
Kuisma M	1995	Resuscitation	10			1	10							1		1		
Graham S	1997	Prehosp Emerg Care	10			1	90											
Suominen	1998	Resuscitation	28			1	96.4											
Perron A	2000	Prehosp Emerg Care	642			184	74.8	505	163	68	81	2	98	7			7	
Nagele P	2000	Anaesthesist	2		9.5	0	100											
Young	2004	Pediatrics	118			6	95											
Lee C	2005	Resucitation	1		1	1	0							1	1			
Patterson M	2005	Pediatric Emergency Care	59	39/20	5	0	100											
Crewdson K	2007	Resucitation	80			7	91.3	50	4	92	7	0	100	3	3			
Lin Y	2007	Resucitation	56	34/22	11	1	98.2	53	1	98.2	3	0	100					
Murphy	2009	J of pediatric surg	169	70/99	10	28	83.5							29	14	3	9	2
Capizzani	2010	J of pediatric surg	30	21/9		6	80							6		2	1	3

Total			1,243		6.92	237	78,2	646	169	73.8	91	2	97.80	47	18	6	18	5

BT: blunt trauma; PT; penetrating trauma; Mort: mortality. Good, Mod, Poor and Veget indicate good, moderate, poor and vegetative outcomes.

In 12 mixed studies (*n *= 229 patients) the mean age could be evaluated as an average of 39.0 years and in 6 pediatric studies (*n *= 329 patients) the average age was 6.9 years.

In 17 mixed studies 2,238 patients had an OHCA after blunt trauma, with a total of 74 survivors (mortality 96.7%). In four pediatric studies, blunt trauma was identified in 646 children, with a total of 169 survivors (mortality 73.8%).

Penetrating trauma was identified in 19 studies with a total of 1,891 patients in the mixed population and 69 survivors (mortality 96.4%), and the data were similar to those in three evaluated pediatric studies of 91 children and 2 survivors (mortality 97.8%).

The neurological classification using the Glasgow outcome scale was analyzed with outcome data for 61 patients in the mixed population and 47 in the pediatric population.

The following outcomes were evaluated in survivors: I) good recovery: full neurologic recovery, total 41.7%, 27 mixed-population patients (44.3%) and 18 pediatric patients (38.3%); II) moderate disability: need of personal help, total 13.0%, 8 mixed-population patients (13.1%) and 6 pediatric patients (12.8%); III) severe disability: daily support needed, total 33.3%, 18 mixed-population patients (29.5%) and 18 pediatric patients (38.3%), and IV) persistent vegetative state: total 10.2%, 6 mixed-population patients (9.8%) and 5 pediatric patients (10.6%).

### Statistical analysis

In univariate analyses of the effects on the proportion of survivors using binomial response generalized linear models, we found no effect of study size (*P *= 0.349), but did find an effect of publication year (*P *= 0.008). Furthermore, we found an effect of gender proportion (*P *< 0.001). However, gender proportions were not reported for all studies, and there was a significant effect of reporting on the survivor proportion (*P *< 0.001). Therefore, we decided to adjust for year of publication, but not gender proportion in the following analyses. When considering only the studies on adults, the estimated proportion of survivors for the reference year 2010 was 8.71% (95% CI 7.40 to 10.24). For each year prior to 2010, the estimated log-odds for survival decreased by 0.022 (95% CI 0.038 to 0.006). For example, the predicted proportion of survivors for 2000 was 7.13%. When jointly analyzing the studies on adults and children, the proportion of survivors for the children (reference year 2010) was estimated to be 17.8% (95% CI 15.1% to 20.8%). The difference between the pediatric and adult proportions of survivors was significant (*P *< 0.001).

Because of the small number of studies with data available on age and mechanism, these two factors could not be analyzed jointly. We also did not analyze the effect of mechanism separately, as a bias might have been introduced by considering two different sets of studies, one where information on age was available, and a second where information on mechanism was available.

On further investigation the mixed population in the European trials, where mainly physicians and EMS were at the trauma scene, were separated from studies performed in other countries where the trauma response was mainly performed only by EMS. The rate of survival was significantly higher in the European studies (*n *= 1,448 patients from 11 studies, mortality 94.1%) compared to the predominantly American evaluations (*n *= 3943 patients from 39 studies, mortality 96.1%) (*P *= 0.0013).

In the pediatric group the survival rate in the predominantly American studies, in which treatment was mainly performed by EMS at the trauma scene (*n *= 1,123 patients from 9 studies, mortality 79.7%), was significantly lower compared to the European investigations where treatment was mainly administered by physicians at the scene (*n *= 120 patients from 4 studies, mortality 92.5%) (*P *= 0.0012).

### Available evidence

No controlled or prospective studies were identified that fulfilled the inclusion criteria for our systematic review. Therefore, no studies were assigned to evidence level I, II or III. According to the level of evidence rating, all studies included were level IV studies.

### Outcome parameters

Only four studies provided an average injury severity score (ISS) and only one reported the time for the beginning of basic and advanced trauma life support. The average time of CPR at the scene and during transport was evaluated in three studies. Three other studies presented data for the average time until hospital admission.

## Discussion

Injury is the leading cause of death in industrialized countries between the ages of one and forty-four years [7,25,26]. Survival after traumatic OHCA is rare, even with maximal resuscitative efforts. There are recommendations in the literature, at least for adults, mandating the emergency team to judge whether to withhold or terminate resuscitation efforts in adult TCPA. Moreover many factors discussed during this decision-making and have been sparsely investigated or are based on marginal or no scientific evidence (such as age, trauma mechanism, time of arrest until start of life support, blood loss and ethical aspects). In order to summarize the available scientific data for some of the above mentioned criteria, the present systematic review was performed.

The aim of the study was to describe the mortality rate after traumatic OHCA in a predominantly adult population and in children taking into account the trauma mechanism and the neurological outcome of survivors on discharge from hospital.

The approach of the present study was also to include case series and studies with a lower level of evidence, because analyses of solely high-quality studies are impossible.

The American College of Surgeons Committee on Trauma suggests withholding resuscitation efforts in any trauma patient found 'apneic, pulseless, and without organized electrocardiographic activity or other sign of life such as spontaneous movements or pupillary reflexes' [7]. It is argued that the number of salvageable patients is too small, considering the inherent costs and risks of resuscitation efforts. A comprehensive review of the literature, on which the 2003 guidelines are based, demonstrated survival rates of 0% to 3.7%, which is similar to our findings except in the pediatric patients [7]. Recently a study of 294 patients, who suffered prehospital TCPA and met the criteria of the guidelines, showed a survival of only one patient (0.3%) with a Glasgow Coma Scale (GCS) of six and violation of the guidelines resulted in six resuscitated patients in a neurologically devastated condition [13]. David *et al. *found in a prospective study that survival and neurological outcome after OHCA did not differ between trauma and medical patients and they suggested that under the supervision of senior physicians, active resuscitation after traumatic OHCA is as important as in medical patients. Moreover they agreed with the findings of Martin *et al. *[6], who suggested that the initial rhythm of the heart (pulseless electrical activity) in trauma is probably not a robust predictor of patient outcome and came to the conclusion that trauma patients in cardiac arrest need active resuscitation in the field until they regain cardiac activity instead of quick transportation to a trauma center, especially if the transport will last for > 15 minutes [7].

On the other hand, there are studies that impressively show survivors being resuscitated against the guidelines of the National Association of Emergency Medical Services Physicians and the American College of Surgeons Committee on Trauma (NAEMSP/ACS-COT), although they had met criteria for non-treatment according to the guidelines [11]. Pickens *et al. *concluded that the prehospital clinical assessments are not reliable for the triage of TCPA patients and that they should be transported to the ED for further evaluation and care [11]. This article described several guideline deviations in traumatic OHCA patients. If EMS personnel had adhered to the guidelines, 111 out of 138 transported patients should not have been transported to the ED and even more striking, 13 out of 14 survivors should not have been resuscitated. Most of the 14 survivors described in that study breached one of the time-related guidelines [11]. The outcome was relatively good with a discharge GCS for the survivors of 13.4 (SD 3.1) with 64.3% (*n *= 9) having the maximum score of 15. Moreover Pickens *et al. *suggested that triaging TCPA patients in the ED would not generate large expenses or lead to lengthy hospitalizations and he thereby attributed the cost associated with trauma resuscitations as one of the reasons for the creation of the guidelines.

The mortality with a penetrating trauma mechanism (96.4% in the mixed population and 97.8% in the pediatric group) versus a blunt trauma mechanism (96.7% in the mixed population and 73.8% in the pediatric group) was significantly different in the pediatric group in our systematic review.

The literature is split on this issue, as several authors have not found any significant difference [3,5,8,11,27], while others have found higher survival among victims of penetrating trauma [28]. Data from Stockinger and McSwain showed a significant survival benefit for victims of blunt trauma [28] and in a study of TCPA patients in the ED, Cera *et al. *identified 13 out of 15 survivors with blunt trauma (*P *= 0.06) [12]. However the penetrating injuries themselves are associated with different outcomes. Durham identified a survival rate of 15.2% in patients sustaining stab wounds compared to 7.3% in patients with gunshot wounds [29].

The guidelines determined that 'penetrating trauma, particularly if isolated to the thorax, has a better prognosis than blunt or multisystem penetrating trauma. Survival from cardiopulmonary arrest due to blunt trauma is grave indeed, likely due to the multisystem nature of the injuries sustained.' [7]. In our analysis the overall survival rate in the mixed population was nearly the same at 3.3% in victims of blunt trauma and 3.65% in victims of penetrating trauma.

The mentioned guideline should not be applied to children since the literature suggests improved survival for children, and moreover, supporting evidence is lacking [30]. There are no specific guidelines for the management of children in cardiac arrest secondary to trauma.

In the evaluated results for children a significant difference was identified between the survival rates of 26.2% in blunt trauma and 2.2% in penetrating trauma. With the knowledge of these data this conclusion of the guidelines probably must be revised in the future.

No evident reason could be found for the relatively high survival rate in children with blunt trauma. Perron concluded in his study that because of the potential that cardiopulmonary arrest in a pediatric patient may not be a result of exsanguination but of a primary respiratory cause, the injured patient having arrested in the prehospital setting may be a different entity to an adult patient in a similar circumstance and warrants a more aggressive initial resuscitative effort [20]. Among children, respiratory compromise is the most common cause of cardiac arrest [31,32]. Previous work has suggested that cardiac arrest in children with trauma is often caused by bleeding or prolonged hypoxemia [16]. Hypovolemic cardiac arrest is often associated with a poor outcome [16,33-35]. The high rate of bleeding associated with hypovolemic arrest in the pre-hospital phase is unlikely to respond to conventional CPR. In contrast, when apnea precedes cardiac arrest, provision of adequate oxygenation may restore spontaneous circulation.

After applying inclusion and exclusion criteria, a total of 47 studies reporting mortality of 5,391 patients after traumatic OHCA were included in the present analysis. The patients' characteristics appear representative for what has been described concerning gender, population and age. Male patients are more likely to be involved in trauma as is the case in the general population. These observations correlated with what has been reported by several review articles earlier.

The vast majorities of the studies identified were case series without a control group, and therefore represent a low level of evidence. No single study with a level of evidence I, II or III was included, so all studies had level IV evidence, which displays insufficient overall evidence. Furthermore, the quality of the investigated published data was relatively poor. Most of the studies found had to be excluded because the reason for resuscitation was non-traumatic. Even though there is so little evidence present in the literature it was surprising that 59 studies had to be excluded because they comprised suggestions on therapeutics and comments, 54 were review articles and 10 were different guidelines. Unfortunately, in 37 studies, patients were not analyzed separately according to whether resuscitation was performed due to traumatic or non-traumatic causes. In the extracted 47 studies important information was missing. For example, the distribution between the sexes was listed in 20 studies only, and the average age of the population was given in 18 studies only.

Against the aim of the authors, some points could not be addressed by the present review. Further limitations are that almost no information was available about relevant parameters in the analyzed studies, such as the period of time before basic or advanced life support was performed; the length of time that CPR was performed until the return of spontaneous circulation; whether CPR was performed at the scene or during the transport; the frequency of the need for CPR until reaching the ED; the length of time taken for transport to hospital and the time period between occurrence of the accident and arrival at the ED. The authors believe that these are also important variables which could have a more or less important impact on survival.

Significant lower mortality was identified by separating the results of the mixed population in European trials, where patients were mainly attended by physicians at the trauma scene compared to the mainly American studies, where EMS performed the trauma response. The biggest European study identified a mortality of 92.5% in 909 evaluated patients [35]. On the other hand there are also huge American studies with mortality between only 91% and 92% [11,29]. In the pediatric group mortality was significantly less in the American studies with one study including 642 pediatric patients with an evaluated mortality of only 74.8% [20]. The biggest European study included 80 patients with an investigated mortality of 91.3% [33]. The potential reasons for the lower mortality could be multiple and should be investigated by further studies. Whether the presence of a physician potentially affects mortality cannot not be proved using the available investigated populations and data.

## Conclusions

With regard to the fact that in this field scientific evidence is very low, further high quality and multi-center studies should be demanded. Results could help in the decision-making of medical teams caring for traumatized patients in the initial out-of-hospital phase when TCPA occurs. Large national registers like the German Trauma Register have played an important role in answering such questions [36] and can continue to do so in the future.

Nevertheless, since the aim of the authors was to describe the literature available on survival and clinical outcome following OHCA caused by trauma, and to draw the best possible conclusion based upon the evidence available, even small studies have not been excluded, but have been analyzed for the most important findings.

However, medical personnel caring for patients with a traumatic OHCA are, and always will be, facing an ethical conflict, and must come to a decision within only a few minutes about when and if to stop resuscitative efforts, knowing about a high percentage of mortality and probably a poor neurological outcome for survivors. But as shown above, more than half of the survivors (adults and pediatric patients) have only moderate disabilities or even gain full recovery of neurological function.

Survival rates can help in the creation of guidelines and standardized decision-making, but after all, the decision is still dependent of the ethical human factor and from persons who have to face every situation based on its own individual criteria, which could influence the decision- making. Therefore, this meta-analysis could support recommendations for future guidelines.

However, evidence based support for the important decision about whether or not to resuscitate a patient in preclinical TCPA is sparse. Thus, the authors hope to encourage all medical disciplines involved in the treatment of such patients to perform further clinical research in this field to obtain a higher level of evidence.

## Key messages

• Children have a higher chance of survival after resuscitation of an OHCA compared to adults, but tend to have a poorer neurological outcome on discharge from hospital.

• Long-term survival is significantly different with 3.3% in a mixed adult/child population and 13.6% in a pediatric population.

• Survival after blunt trauma is significantly higher in the pediatric group.

• Long-term survival is good and moderate neurological recovery is reported in 57.4% of all survivors in a mixed adult/child population and in 51.1% of a paediatric population.

• Starting CPR in trauma patients may be worthwhile and trauma management programs should be discussed critically.

## Abbreviations

CI: confidence interval; CPR: cardiopulmonary resuscitation; ED: emergency department; EMS: emergency medical services; ISS: injury severity score; OHCA: out-of-hospital cardiopulmonary arrest; TCPA: traumatic cardiopulmonary arrest.

## Competing interests

The authors declare that they have no competing interests.

## Authors' contributions

JZ and PS made substantial contributions to the design of the study and the acquisition of data and analysis of data. All authors JZ, AM, TH, JB, NS, PS have made substantial contributions to all of the following: the conception of the study, interpretation of the data drafting the article or revising it critically for important intellectual content. All authors read and approved the final manuscript.
